# Progress in Medicine

**Published:** 1900-08-25

**Authors:** 


### Progress in Medicine.

### NEUROLOGY.

*(Continued from page 337.)*

**Migraine.**—Spiller[12] records two cases which appear to be connecting links between migraine and epilepsy. He remarks that we can distinguish the following forms: Attacks of migraine associated with nausea and vomiting; this form is known as simple migraine and usually remains unaltered during the life of the patient. (2) In other cases visual disturbances (hemianopsia, scintillating scotoma, amaurosis, &c.) are associated with the migraine, and the disease is then known as ophthalmic migraine. (3) When paralysis of the ocular muscles occurs with the migraine the disease is described as ophthalmoplegic migraine. (4) Migraine, especially the ophthalmic form, is related to epilepsy, and the attacks of migraine may precede for many years the convulsive attacks of epilepsy. (5) In some cases epilepsy appears in the form of one or more of the disturbances seen occasionally with migraine, and later convulsions develop. It matters little whether we regard these as abortive cases of migraine that later become associated with epilepsy, or as abortive forms of epilepsy (sensory epilepsy) in which the convulsions later become apparent, provided we recognise a relation between some forms of migraine and epilepsy. In the first of the cases recorded there were recurrent attacks of unilateral headache, with transitory paresis and paræsthesia of the opposite side of the body, and paraphasia. In the second case there were recurrent attacks of, at first, numbness of the right side of the tongue and inability to speak; and, later on, additional numbness of the right arm and leg. The development of the numbness followed a definite course, first the tongue, then the right upper limb, and finally the right lower limb. There was no loss of consciousness, and no convulsion.

**Family Periodic Paralysis.**—Mitchell[13] has recorded a careful study of a case of family periodic paralysis—a condition, fortunately, of the utmost rarity. The case, like all the cases observed, presented the following features: A paralysis of the voluntary muscles, usually total except for the head and face muscles, with loss of reflexes, with absence of electrical reaction, with no mental or sensory disturbances, and occurring periodically at somewhat regular intervals. There was a well-marked history of similar attacks in various relations on the mother's side. The attacks began, in the patient's thirteenth year, suddenly and without known cause; he awoke one morning and found he could not move hand or foot; he was able to turn his head from side to side, but could not raise it from the pillow. There was no pain, or loss of sensation, or impairment of function of any kind. About two months after the first attack another occurred, and for three or four years they returned at intervals of about two months, with a duration of about 48 hours, nearly always coming on in the night. No possible cause can be assigned in his habits or mode of life. Except that when they pass he finds himself a little sore and stiff for a few hours, he cannot see that he is otherwise than perfectly well in the intervals. After four years or so the interval shortened, and at the time when the case was investigated was about a week. Nothing about the patient suggested the slightest possibility of a hysterical element. He was a normal healthy boy, in good physical condition, and much disgusted by the enforced idleness of his disablement. Breathing in the attacks was somewhat shallow, and appeared to be altogether diaphragmatic in character. He was unable to cough, nor did he sneeze on irritation of the nerves. He could swallow and speak, the mind was unaffected, and he said he had no pain—only the discomfort of lying absolutely in one position. Sensation was perfect; all reflexes except the reaction of the pupil were lost. There was total abolition of reaction to any form of electrical stimulation. The tongue was somewhat coated, and the breath had a heavy smell. A few hours after the paralysis had disappeared the muscles responded normally to faradic and galvanic currents.

The first view which suggested itself to account for these extraordinary attacks was that the trouble had an autotoxic origin. The blood, fæces, and urine were therefore examined in the most careful and detailed manner, both during the attacks and in the intervals. The most minute study, however, revealed no fact of importance in any of them.

Strychnine, bromides, internal antiseptics were all useless in treatment. The affection presents some analogy to migraine, which is also a disease in which family tendency is strongly evinced, which usually begins at about the age of puberty, and lessens or disappears in middle life, which occurs paroxysmically and often at fairly regular intervals. The heavy breath, coated tongue, comparative suppression of urine during the attack, and an unusual flow after, all carry out the analogy to migraine.

The essential differences between the diseases are that the manifestations in the one case are largely cerebral, whilst in the other they appear in the lower segment of the motor path.

The disease is remarkable in this, that it is clinically a most distinct entity, that all observers agree in their statements of the vast majority of symptoms, and that up to the present time the causation and pathology of the affection remain in absolute darkness.

Putnam[14] has recorded a very similar case. In discussing it, he remarks that the condition is essentially one of periodic inhibition of the voluntary muscles. As to the cause of this, he thinks that the long and hitherto barren hunt after "poisons" is unfruitful, and that it is showing too little respect for the subtle processes of inhibition in their relation to states of health to suppose that the blood and urine should contain substances incompatible with the life of cats and mice. In the ordinary condition of healthy muscles the resultant of the two opposing forces of relaxation and contraction is shifted somewhat to the motor side, and thence the tonus. Yet one may easily conceive that under slight modifications of nutrition or growth of the nervous system its reaction even towards normal fluids should be so altered that the resultant of two forces should be shifted periodically towards the side of relaxation.

Another case of family periodic paralysis is recorded by Crafts,[15] in which the clinical phenomena were very similar. The special interest of the case lies in the fact that Irwin, who examined the urine, blood, and fæces, claims to have isolated from the fæces a toxic product which, when injected into rabbits or guinea-pigs, produced motor paralysis similar to that in the patient, and disappearing in the same length of time.

[12] Spiller, Amer. Jour. Med. Sci., Jan , 1900. [13] Mitchell, Amer. Jour. Med. Sci., Nov., 1899. [14] Putnam, Amer. Jour. Med. Sci., Feb., 1900. [15] Crafts, Amer. Jour. Med. Sci., June, 1900.

